# Large-scale cross-sectional online survey on patient-neurologist communication, burden of disease assessment and disease monitoring in people with multiple sclerosis

**DOI:** 10.3389/fneur.2022.1093352

**Published:** 2023-01-04

**Authors:** Monika Christ, Katrin Schuh, Antonios Bayas

**Affiliations:** ^1^Department of Neurology, Faculty of Medicine, University of Augsburg, Augsburg, Germany; ^2^Novartis Pharma GmbH, Nuremberg, Germany

**Keywords:** multiple sclerosis, survey, patient communication, magnetic resonance imaging, digital technology

## Abstract

**Background:**

Management of multiple sclerosis (MS) requires a high level of communication between health care professionals (HCPs) and people with MS (pwMS) including profound investigation and discussion of symptoms to identify therapeutic needs. For treatment decisions, monitoring of disease activity is important, in this respect self-monitoring devices and apps, as well as magnetic resonance imaging are important tools.

**Methods:**

MS Perspectives is a cross-sectional online survey conducted in Germany which was designed to collect data, among others, on the communication between pwMS and HCPs regarding treatment goals, symptom assessment, usage of devices and apps to self-monitor health functions, as well as to identify patients' attitude toward the role of magnetic resonance imaging (MRI). Between December 2021 and February 2022, 4,555 pwMS completed the survey.

**Results:**

In total, 63.7% of participants reported that treatment goals have been discussed with their HCPs. Symptoms worsening in the past 12 months independent of relapses was more often reported by pwMS than inquired by HCPs, according to patients' report. Devices or apps for health monitoring were used by less than half of participants. Frequency of MRI controls was much lower in participants with longer compared to shorter disease duration (47.5 vs. 86.3%). The proportion of patients with annual or semiannual scans was highest among pwMS receiving infusion therapy (93.5%), followed by oral medication (82.5%) and injectables (73.4%), and lowest for pwMS without immunotherapy (58.2%).

**Conclusion:**

MS Perspectives identified a rather low patient involvement regarding treatment goals and symptom assessment in clinical practice. Regarding this and our findings for health self-monitoring and MRI usage, strategies for improving patient-HCP communication and disease monitoring may be considered.

## 1. Introduction

Optimal management of multiple sclerosis (MS) requires a high level of communication between health care professionals (HCPs) and patients. Besides established clinical tests, e.g., to assess walking performance, cognition, and depression, a comprehensive patient assessment should necessarily also focus on a profound investigation and discussion of symptoms experienced by patients in their daily life (1). Based on the information obtained, treatment should be reconsidered and adjusted, if necessary. In addition, a discussion on treatment goals can result in setting up expectations agreed on by HCPs and patients also improving treatment adherence (2, 3).

Within the last few years, numerous technical devices and apps have been introduced to support monitoring of MS symptoms and progression (4). However, it is unknown how many people with MS (pwMS) use such devices for health monitoring and if they are part of patient-HCP communication.

Magnetic resonance imaging (MRI) of brain and spinal cord is the most important technique for diagnosing MS and tracking disease evolution. However, the perception of the role of MRI by pwMS and its influence on HCP-patient communication has not yet been addressed at larger scale.

The online survey MS Perspectives, among others, aimed to assess the extent of exploring symptom progression by HCPs, patients' symptom reporting to HCPs, the use of digital tools for monitoring symptoms, as well as whether select symptoms are reported less frequently, and whether specific symptoms may be more likely to trigger actions, including treatment changes. Furthermore, we investigated the perception of the role of MRI for the patient-HCP communication by pwMS.

## 2. Patients and methods

### 2.1. Data collection

MS Perspectives is an online survey conducted in Germany between December 2021 and February 2022 through an online data collection tool hosted by ClinLife^®^ from Clariness. The survey included pwMS in Germany from the age of 18 years up. Details on recruitment, the questionnaire used, as well as demographic and disease characteristics of participants have been published previously (5). Briefly, 4,555 pwMS completed the survey, 69.2% reported to have relapsing remitting (RR) MS, 15.1% secondary progressive (SP) MS, and 7.2% reported to have primary progressive (PP) MS (8.4% did not know their MS subtype). 82.0% were between 26 and 55 years of age, and 85.2% were female. According to either Expanded Disability Status Scale (EDSS) score or self-assessment, 72.3% of the total population were classified as having no or mild to moderate disability, and 27.7% were classified as having marked to severe disability (5).

Here, we report the results of survey questions pertaining to the communication between pwMS and HCPs (questionnaire items no. Q23, Q24a, Q24b, and Q25) (Supplementary Table 1), to issues addressed during neurologist visits (questionnaire item no. Q26), to the usage of devices and apps to self-monitor health functions (questionnaire items no. Q33–Q36), as well as to MRI assessments (questionnaire items no. Q27–Q32). Only participants having answered all questions were included in the analysis.

### 2.2. Statistics

All data was analyzed descriptively. No formal statistical testing for group comparisons was performed. Categorical variables were summarized using frequency counts and percentages.

Subgroup analyses involved comparisons by MS subtype. Furthermore, analyses are presented by disability status determined by EDSS as assessed at the last appointment with the neurologist, or self-assessed disability status (no or mild to moderate disability: EDSS 0-3.5 or no / minimal / moderate disability [unrestricted walking distance]; marked to severe disability: EDSS 4-9.5 or walking distance restricted, but able to walk 500 m without assistance / able to walk 200 m without assistance / unilateral assistance required for 100 m / walking distance restricted up to 5 m, predominantly restricted to wheelchair / predominantly restricted to wheelchair, chair, or bed) (5).

## 3. Results

### 3.1. Patient-HCP communication

**Figure 1:**
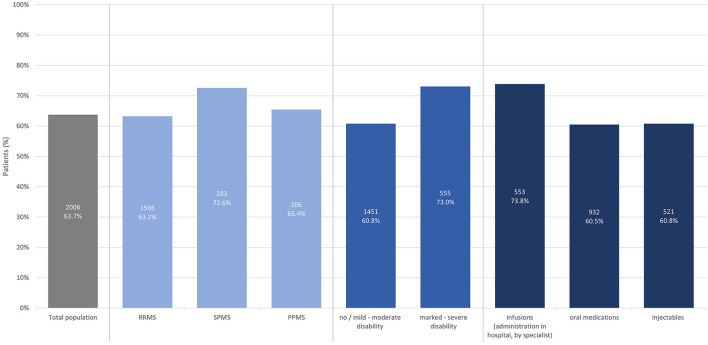
Proportion of patients with immunotherapy reporting that treatment goals have been discussed (total population, by subtype, by disease severity, by treatment modality).

Most participants on immunotherapy (63.7%) reported that treatment goals have been discussed with their HCP. Numbers were slightly higher in SPMS (72.6%), for participants with marked to severe disability (73.0%), and those receiving infusions as immunotherapy (73.8%) (Figure 1).

**Figure 2:**
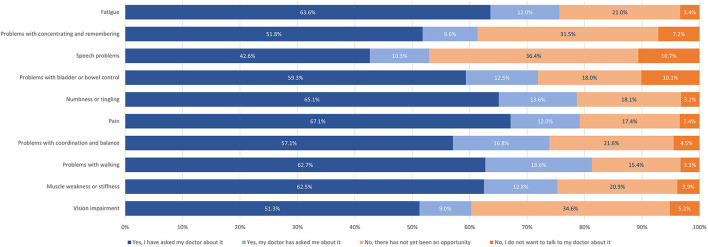
Reporting of symptoms worsened in the last 12 months independent of relapses.

All pwMS with symptoms having continuously worsened in the last 12 months independent of relapses (5) were asked, whether they had talked about it to their HCP and which consequence was drawn, if at all. PwMS reported their symptoms on their own initiative in the majority of cases, a far smaller proportion of symptoms was identified by HCPs' inquiries. The lowest self-reporting rate was observed for problems with speech (42.6%), the highest for pain (67.1%). Symptoms with the lowest rate of HCP-initiated inquiries were problems with vision (9.0%), and the highest rate was observed regarding problems with walking (18.6%). Speech problems (47.1%), vision impairment (39.8%) as well as cognitive symptoms (38.7%) were most frequently not addressed by participants because, as selected, there had been no opportunity or because they did not want to talk about them with their HCP (Figure 2).

**Figure 3:**
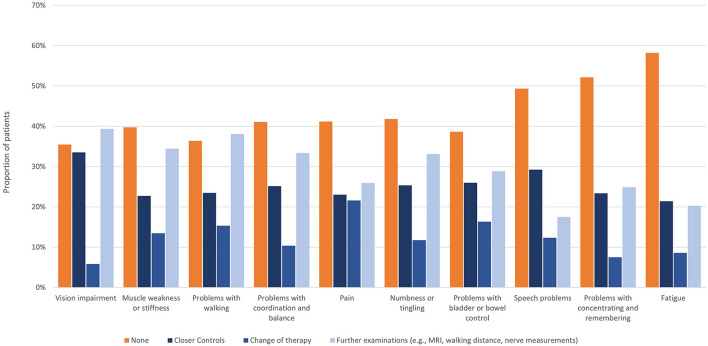
Actions taken regarding reported symptoms.

Fatigue, cognitive problems, and speech problems did not trigger any actions in about half of participants. Symptoms most frequently triggering treatment switches were pain (21.6%), problems with bladder or bowel control (16.3%), and walking problems (15.3%) (Figure 3). The type of immunotherapy (oral, injectables, infusions) had no effect on the rate of symptom self-reporting, nor the actions taken (Supplementary Figures 1, 2).

**Figure 4:**
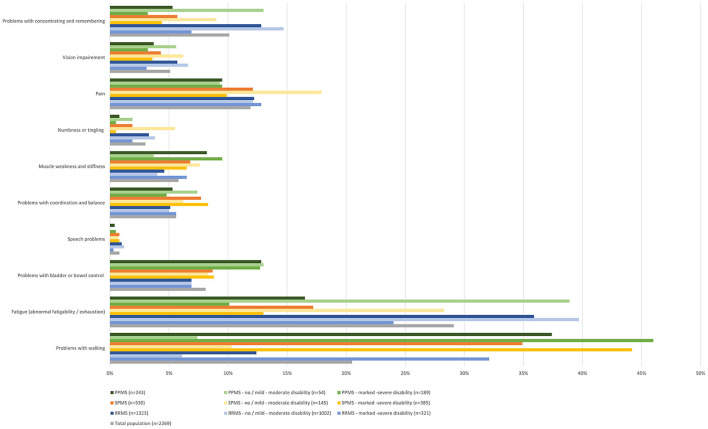
Most disabling symptoms worsened in the last 12 months independent of relapses (total population, by subtype, by disease severity).

Participants were further asked, which symptoms worsening relapse-independently in the past 12 months were most disabling in their daily life. Most disabling symptoms in RRMS were (in descending order) fatigue (35.9%), cognitive impairment (12.8%), problems with walking (12.4%), pain (12.2%) as well as problems with bladder and bowel control (6.9%). In SPMS the most disabling symptoms worsening relapse-independently were problems with walking (34.9%), fatigue (17.2%), pain (12.1%), problems with bladder and bowel control (8.7%) as well as problems with coordination and balance (7.7%). In PPMS most disabling symptoms were problems with walking (37.4%), fatigue (16.5%), problems with bladder and bowel control (12.8%), pain (9.5%), as well as muscle weakness and stiffness (8.2%) (Figure 4).

**Figure 5:**
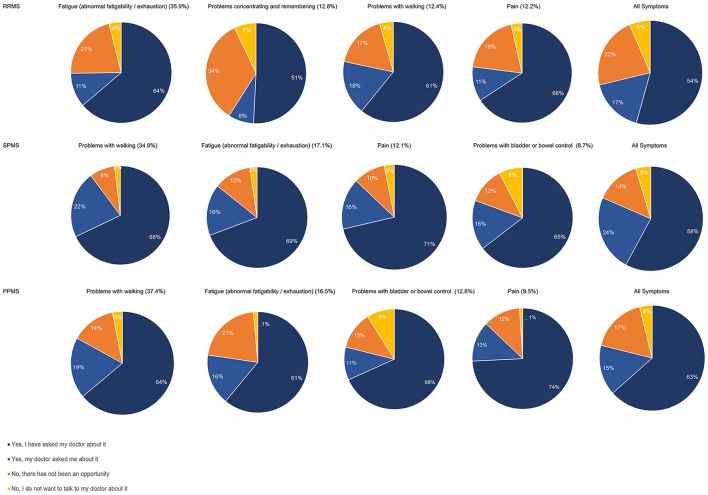
Reporting of most disabling symptoms worsened in the last 12 months independent of relapses by subtype; % in brackets gives the proportion of patients who reported the symptom in the respective subpopulation.

**Figure 6:**
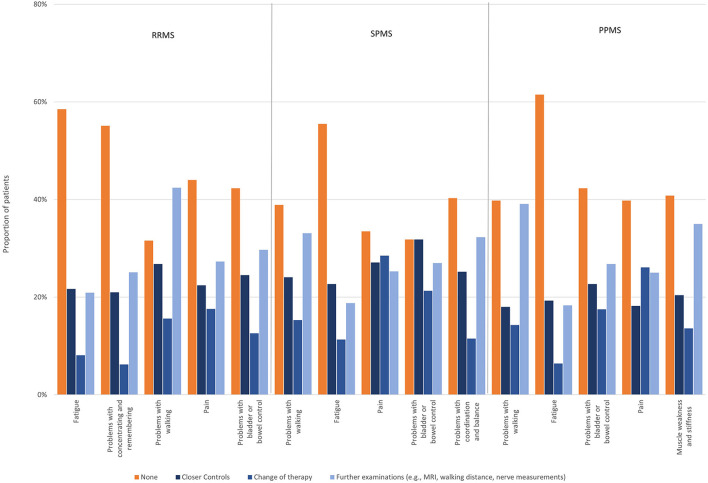
Actions taken regarding most disabling symptoms worsened in the last 12 months independent of relapses by subtype.

Reporting rates (self-reported or HCP-inquiries) of the most disabling symptoms worsening relapse-independently in the past 12 months were, for most symptoms, higher than the reporting rate of all symptoms worsening relapse-independently in the respective MS group. Below-average reporting rates were only observed for problems with concentrating and remembering in RRMS patients (59%), for problems with bladder or bowel control in SPMS (81%) and for fatigue in PPMS patients (77%). Notably, there was a high proportion of RRMS patients who stated that there was no opportunity to report problems with concentrating and remembering (34%). For problems with bladder or bowel control, an over-average proportion of patients with SPMS (8%) and PPMS (9%) reported that they did not want to talk to their physician about this symptom, even though it was reported as the fourth (SPMS) and third (PPMS) most disabling symptom in these patient groups (Figure 5). Most disabling symptoms worsened in the last 12 months independent of relapses most frequently triggering treatment changes were pain (17.6%), problems with walking (15.6%) and problems with bladder and bowel control (12.6%) in RRMS; pain (28.5%), problems with bladder and bowel control (21.3%) and problems with walking (15.3%) in SPMS and pain (26.1%), problems with bladder and bowel control (17.5%) and problems with walking (14.3%) in PPMS. Fatigue was the symptom that most often did not trigger any of the polled actions in all groups [RRMS (58.5%), SPMS (55.5%), PPMS (61.5%)] (Figure 6).

### 3.2. Frequency and types of clinical assessments

**Figure 7:**
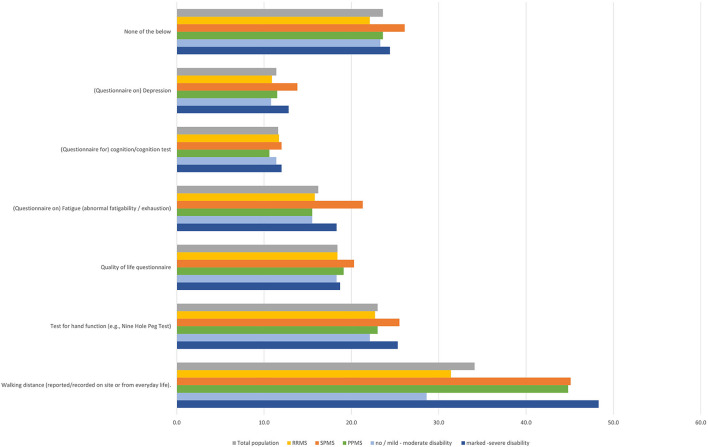
Regular examinations during visits at neurologist (total population, by subtype, by disease severity).

Evaluation of the walking distance was the most frequent examination at the neurologist (34.1% of the total population). For walking distance evaluation, slight differences in frequency by MS subtype were observed, being assessed more frequently in SPMS and PPMS patients compared to RRMS patients (RRMS 31.4%; SPMS 45.1%; PPMS 44.8%). Assessment of fatigue was quite uniform (RRMS 15.8%; SPMS 21.3%; PPMS 15.5%), also for other assessments, no relevant differences were reported regarding MS subtype. Similarly, disease severity had no impact on clinical examinations (Figure 7).

### 3.3. Health self-monitoring

**Figure 8:**
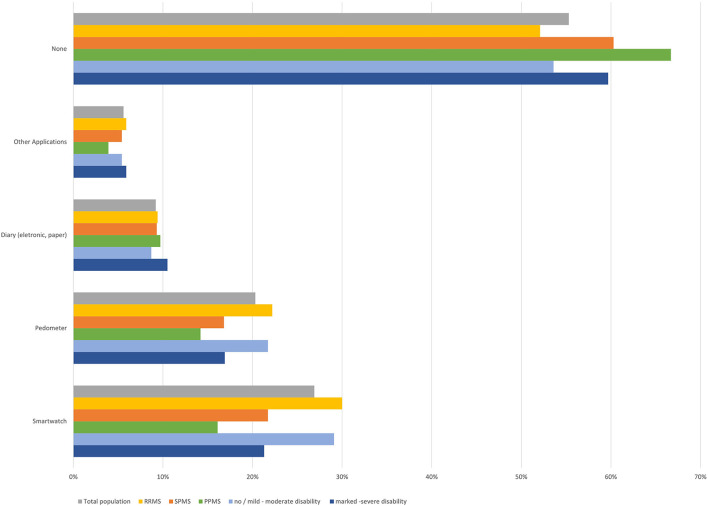
Use of health monitoring gadgets and applications (total population, by subtype, by disease severity).

Health-monitoring devices (smartwatches such as Apple Watch or Fitbit, pedometer, electronic or paper diaries) or (other) apps were used by less than half of participants (Figure 8). Most frequently mentioned “other apps” were Cleo^TM^ and Brisa^®^, both MS-specific apps. Slight differences regarding self-monitoring with pedometers and smartwatches were observed by MS subtype and disability status, with higher proportions in RRMS and participants with lower disability status compared to SPMS and higher disability status (Figure 8). No age-dependent differences were observed, apart from a slightly more frequent use of “other apps” in younger participants (especially those aged 18–25 years; Table 1).

**Table 1:** Use of health-monitoring gadgets and applications by age.

**Age group**	**Smartwatch %**	**Pedometer %**	**Diary %**	**Other apps %**	**None %**
18–25 (*n* = 266)	26.7	22.6	14.7	9.4	49.2
26–35 (*n* = 1,215)	28.6	22.1	9.0	6.5	53.6
36–45 (*n* = 1,405)	28.8	20.8	8.8	5.3	54.7
46–55 (*n* = 1,114)	27.0	18.4	8.7	4.2	56.1
56–65 (*n* = 496)	17.9	17.7	8.3	5.0	62.7
>65 (*n* = 59)	20.3	22.0	11.9	5.1	54.2

n, Number of patients in the category.

78.6% of participants using devices and apps reported to use them on a regular basis, 21.4% intermittently. Most frequently, app functions used were documentation (66.5%) and measurement of body functions (59.1%), followed by memory functions (49.6%) and information search (43.3%). 15.0% of participants reported to use apps for communication and 11.0% for other functions. Regarding the app functions, only slight differences were observed between age groups (Table 2). Only one third of participants (33.9%) reported that app functions have played a role in the communication with their neurologist.

**Table 2:** App functions used by age.

**Age group**	**Documen-tation %**	**Body function %**	**Memory function %**	**Information search %**	**Communi-cation %**	**Other %**
18–25 (*n* = 25)	72.0	56.0	60.0	44.0	8.0	8.0
26–35 (*n* = 79)	69.6	48.1	54.4	39.2	13.9	7.6
36–45 (*n* = 75)	68.0	72.0	46.7	53.3	25.3	9.3
46–55 (*n* = 47)	55.3	61.7	48.9	40.4	4.3	17.0
56–65 (*n* = 25)	64.0	48.0	36.0	28.0	8.0	20.0
>65 (*n* = 3)	100.0	100.0	33.3	66.7	66.7	0.0

n, Number of patients in the category using apps.

### 3.4. MRI

**Figure 9:**
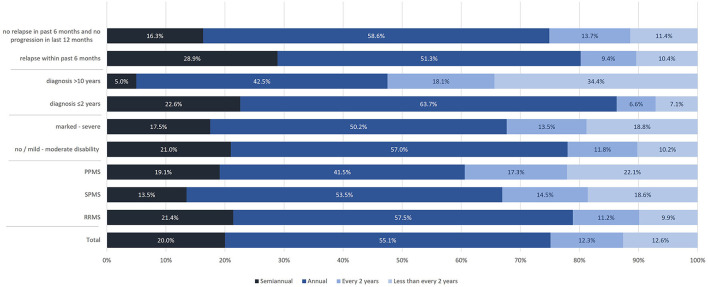
Frequency of MRI assessments (total population, by subtype, by disease severity, by time of diagnosis, by relapse activity/progression).

Most participants had semiannual or annual MRI scans. The proportion of participants receiving semiannual or annual MRI was slightly lower in SPMS and PPMS patients compared to those with RRMS (67.0% and 60.6 vs. 78.9%), in participants with marked to severe disability compared to those with no or mild to moderate disability (67.7 vs. 78.0%), and in participants without disease activity or disability progression compared to those with relapses in the previous 6 months (74.9 vs. 80.2%). The frequency of MRI controls was much lower in participants with longer disease duration compared to those being diagnosed more recently (47.5 vs. 86.3%) (Figure 9).

The proportion of patients with annual or semiannual scans was highest among those receiving infusion therapy (93.5%), followed by patients with oral medication (82.5%) and injectables (73.4%). Lowest rates were observed for patients without immunotherapy (58.2%). This finding was independent of the disease course (RRMS: no immunotherapy 63.6%, injectables 75.3%, oral 84.3%, infusions 94.7%; SPMS: no immunotherapy 52.0%, injectables 65.2%, oral 75.7%, infusions 90.8%; PPMS: no immunotherapy 44.0%, injectables 57.7%, oral 70.9%, infusions 88.9%).

In most participants receiving MRIs, contrast agents were applied (always: 47.1%; frequently: 32.6%). Also, participants without relapse activity in the past 6 months and disease duration >1 year had contrast agents applied regularly (Table 3). Only 20.2% reported contrast agents having never been applied, independent of disability, equal in RRMS and PPMS, but slightly lower in SPMS.

**Table 3:** Frequency of contrast agent use for MRI.

**Patient group**	**Always %**	**Frequently %**	**Never %**
Total population (*n* = 4,555)	47.1	32.6	20.2
RRMS population (*n* = 3,151)	45.8	33.0	21.2
SPMS population (*n* = 690)	50.9	35.1	14.1
PPMS population (*n* = 330)	48.8	29.7	21.5
No / mild to moderate disability (*n* = 3,293)	46.5	32.9	20.6
Marked to severe disability (*n* = 1,262)	48.8	32.0	19.2
No relapse activity, disease duration >1 year (*n* = 393)	49.6	34.6	15.8

n, Number of patients in the category; RRMS, relapsing-remitting multiple sclerosis; PPMS, primary progressive multiple sclerosis; SPMS, secondary progressive multiple sclerosis.

Most participants reported to consult the same radiologist for MRI assessments (80.1%), in the majority of participants MRI scan results are discussed with the HCP (90.3%). In total, 70.0% of participants selected that MRI monitoring is important for them (e.g., to provide security regarding stable disease). Only 17.1% stated that it is not important (e.g., because it is just an ‘image' that has no meaning without symptoms). 12.9% had no opinion toward the importance of MRI monitoring. Almost half of participants (46.4%) wanted to learn more about the role of MRI in their condition, whereas 31.8% did not, and 21.8% had no opinion on that.

## 4. Discussion

MS Perspectives represents a large-scale cross-sectional study. Here we report findings on the communication between pwMS and HCPs, frequency and relevance of clinical and MRI assessments, as well as utilization of health self-monitoring.

Surprisingly, only around two thirds of pwMS with immunotherapy reported that treatment goals have been discussed with them. The situation is somewhat better for SPMS, for pwMS with higher disability and infusion therapy, in the latter group possibly related to more frequent visits. However, also in these subgroups every fourth participant reported to be uninformed about treatment goals. In a study developing a preference assessment tool, 87% of pwMS wanted to discuss treatment goals and priorities with their HCPs, but 37% also reported that they do not routinely share their individual goals and priorities (6). Another study focusing on challenges faced by HCPs in patient management found that 39% of neurologists reported no or low skills to integrate patients' individual goals into treatment recommendations (7). Based on that, an inadequate or lacking communication between patients and HCPs seems to originate from both, the patients' and HCPs' sides. In our study however, only the patient's perspective has been investigated. Nevertheless, this finding is of high relevance, as clear treatment goals and shared decision making have been identified as relevant factors for treatment satisfaction and adherence (8). However, it has to be kept in mind that the preferred level of involvement in making medical decisions may vary between pwMS (9).

Our survey also investigated a potential under-reporting of symptoms by pwMS. Regarding symptoms worsening independent of relapses, the highest patient-initiated reporting rate was found for pain (67.1%), followed by sensory disturbances, fatigue, walking problems, and motor symptoms. Of note, reporting of motor symptoms exceeded cognition-related impairments, although both were stated almost equally to be the most disabling symptom especially in RRMS patients. Other symptoms frequently affecting quality of life, like voiding disorders, were less frequently reported. Our survey does not allow to ascertain how often HCPs would or would not have inquired select symptoms, if not reported by participants themselves. However, our findings indicate that there is a clear under-reporting of MS symptoms in the patient-HCP communication requiring improvement, ideally by using a structured questionnaire and encouraging pwMS to report symptoms. This may improve early identification of relapse-independent progression and potentially prompt adjustments of immunotherapy. Furthermore, there is the danger that under-reporting of symptoms, among them pain, fatigue, and walking problems, may also result in both pharmacological and non-pharmacological undertreatment of various symptoms, non-uniformly distributed in different MS types. Moreover, the initiation of rehabilitation, an important backbone of MS treatment in patients with all types of MS, intended to improve functional outcomes and quality of life (10), may adversely be affected.

In a survey conducted among over 900 pwMS, a high number reported that they are uncomfortable speaking about symptoms, especially if intimate problems like sexual (54%), bladder and bowel dysfunctions (28%) or mood swings (26%) are concerned (11). Feeling comfortable speaking to their neurologists was also associated with higher treatment satisfaction in the same study, highlighting the relevance of a trusting communication (11). The “MS in the 21st Century Steering Group” including HCPs and pwMS, identified time constraints as another issue in clinical consultation. Both HCPs and pwMS reported to be dissatisfied with the limited time (12). This may also contribute to an under-reporting in daily clinical practice but was not assessed in our survey.

Symptom assessments seem to be independent of the severity and course of the disease. Only assessments of walking distance and fatigue appeared to be more frequent in SPMS patients.

Of note, in < 40% of participants only, reported symptoms triggered actions that could be selected in the survey, i.e., closer controls, treatment change, further examinations, most often concerning vision impairment and walking problems. However, it has to be pointed out that from the data obtained, symptom severity and whether actions taken were based on a single or various symptom(s), remain obscure. Moreover, it remains open whether reporting select symptoms triggered any actions or considerations, which have not been perceived by the participant. Overall, treatment changes were less common than other reactions that could be reported, but somewhat more frequent in participants with SPMS. However, the survey did not inquire, if treatment changes concerned immune or symptomatic treatments or if rehabilitative training was initiated. Furthermore, it is not known, if further examinations being triggered had then an impact on treatment decisions.

In summary, our data indicate that many symptoms are not discussed with HCPs, and even if they are, it remains without consequences in a relevant number of pwMS. However, it has to be noticed that our results may be biased since we only assessed the patients' perspective. Communication and reacting to reported symptoms seem to be more prevalent in SPMS and pwMS receiving infusions.

In a complex disease like MS, the characterization and assessment of MS symptoms is challenging and difficult to achieve in clinical practice. Within the last few years, digital technology devices have been introduced that can contribute by collecting so-called digital biomarkers (4). This is especially important in progressive MS, where fluctuating symptoms also have to be considered and history taking is often difficult. In our survey, devices and apps for health self-monitoring were used by less than half of the participants, with smartwatches being used most often. Using devices and apps was not clearly dependent on age, which may be due to the fact that participants in this online survey are more prone to digital technologies irrespective of age. In an analysis of a sample of US adults, older individuals were less likely to use health apps. One third of smartphone or tablet users had a health app installed on their device and half of them reported that apps have helped them to achieve a health-related goal (13). Among participants in the “North American Research Committee on Multiple Sclerosis” (NARCOMS) registry, a survey regarding the use of health apps was published in 2019. Of those who reported using a smartphone or tablet, 46.2% used a mobile health app, comparable to our findings. Factors associated with a higher likelihood of reporting the use of mobile health apps and with perceived benefits of using these apps included younger age, online survey response, having comorbidities, as well as higher income and education levels (14). Participants in our study using devices and apps, usually use them frequently, with about two third using them for disease documentation or assessment of vital functions. One third of participants reported that app functions and documentations were part of the discussion during their neurological consultation. This is in line with observations from patients in primary care. In a survey amongst primary care physicians, only one quarter reported that at least some of their patients had sent or brought their self-documented health data (15).

Implementing self-monitoring data from devices and apps in the assessment of the disease evolution could improve disease management, facilitate early identification of progression and the potential need for treatment adaption. Notably, the indication for initiating or improving symptomatic and prompting rehabilitation treatment may become evident. Especially in cognitively impaired pwMS, real-time documentation of symptoms in apps including onset, severity, and resulting impairments could improve and facilitate reporting of symptoms. Furthermore, wearable sensors including commercially available fitness trackers could for example improve long-term tracking of physical activity (16–18), which is also important for aging pwMS. Brichetto et al. have proposed a patient-centered model of disease management that involves portable / wearable devices to self-monitor the health status, to stimulate lifestyle adaptions, and to induce intervention from HCPs, either automatically or patient-initiated (18). However, in order to draw relevant conclusions, apps and devices have to produce reliable data. For example, as a limitation in pwMS, accelerometers or pedometers have been found to be inaccurate with an EDSS > 6 (18). An increasing and widespread use of digital tools and technology and the potential for improving patient care using those data, requires the availability of appropriate health care resources, requiring, among others, technical equipment, and staffing. However, the availability of such resources may greatly vary between different health care sites, e.g., hospitals, outpatient departments or medical practices, and regions. This may therefore result in an unequal distribution of the potential to use these technologies.

The value of MRI in pwMS for monitoring purposes is well-established (19). The difficulty comparing serial MRIs due to different scanners and image acquisition protocols used is a major concern in clinical practice resulting in a reduced relevance of this important insight in disease evolution. For this, in control scans the use of the same scanner and image acquisition protocols is highly recommended. In our survey, most participants (80.1%) reported to consult the same radiologist for all MRI assessments, a high number that we did not expect, based on our experience in a tertiary referral university hospital. On the other side, in every fifth patient this should be optimized to grant comparability of serial MRIs in order to draw meaningful conclusions.

As expected, MRI examinations were less frequent in pwMS with longer disease duration, possibly because of stable patients in the follow-up. Also in progressive forms of MS, MRI controls were reported to be less frequent that may be explained by the fact that conventional MRI sequences only partly correlate with and predict disability progression in people with progressive MS (20), likely prompting neurologists to refrain from more frequent controls. Surprisingly, despite recommendations by the German Competence Network MS (21) and the ‘Magnetic Resonance Imaging in Multiple Sclerosis study group–the Consortium of Multiple Sclerosis Centers–and North American Imaging in Multiple Sclerosis Cooperative MRI guidelines working group‘ (MAGNIMS–CMSC–NAIMS) consensus (19) regarding the select use of intravenous gadolinium-based contrast agents, contrast agents were reported to be applied very frequently (‘always‘ in 47.1%, frequently in 32.6%) with no or minor differences between disease courses and disability status.

As expected, the proportion of pwMS with regular MRI follow-up was highest among those receiving infusion therapy (93.5%), followed by oral medication (82.5%) and injectables (73.4%) with lowest rates for pwMS without immunotherapy (58.2%). This observation was independent of the disease course. Our findings may reflect the need for more frequent MRI controls due to higher disease activity and also increased pharmacovigilance in pwMS receiving infusion therapies, e.g., natalizumab, or higher efficacy oral medication compared to pwMS treated by injectables (beta-interferons or glatiramer acetate). Of note in the survey we did not assess specific DMTs, approved DMTs at the time of the survey were: alemtuzumab, beta-interferons, cladribine, dimethyl fumarate, diroximel fumarate, fingolimod, glatiramer acetate, mitoxantrone, natalizumab, ocrelizumab, ofatumumab, ozanimod, ponesimod, siponimod, teriflunomide. Therefore, oral and injectable therapies reported may have been lower or higher efficacy treatments.

What we regard as a favorable finding is that MRI results were reported to be discussed in most participants.

Despite data originating from a large and representative MS cohort (5), the study has some limitations. First, no validated questionnaire was used, and second, no formal hypothesis testing was applied. The results therefore are rather hypothesis-generating than confirming and need to be interpreted with caution. Third, web-based medical surveys are prone to a participation bias toward participants with higher level of education and better health state (22). Finally, a potential bias has to be taken into consideration as only responders who answered all questions were included and thus participants with higher self-motivation and disease awareness might be overrepresented (5). Furthermore, due to the nature of our study, HCPs were not surveyed resulting in a potential bias especially regarding patient-HCP communication that has to be noted when interpreting results.

In summary, MS Perspectives gives an important insight in the patient-HCP communication, according to the study design from the patients' view, indicating that identifying MS symptoms as well as the discussion of treatment goals may be improved. Second, health self-monitoring apps and devices are used only by a minority of patients. Given the important information provided by these technologies and the facilitation of the assessment of disease evolution, strategies to enhance their use by more pwMS should be promoted. Third, performing serial MRIs at the same scanner and with the same acquisition protocol, may be improved, at least in a minority of participants. Regarding the reporting rates for applying contrast agents, the use of it should be critically considered based on published recommendations.

## Data availability statement

The raw data supporting the conclusions of this article will be made available by the authors upon request, without undue reservation.

## Ethics statement

Ethical review and approval was not required for the study on human participants in accordance with the local legislation and institutional requirements. Written informed consent for participation was not required for this study in accordance with the national legislation and the institutional requirements.

## Author contributions

MC was responsible for the conceptualization of the manuscript, analysis, data curation, visualization, and writing of the manuscript. KS was responsible for the conceptualization of the study and the manuscript, study methodology, data curation, funding acquisition, project administration, and as well as reviewing and editing the manuscript. AB was responsible for the conceptualization of the study and the manuscript, study methodology, data curation, analysis, visualization, and writing of the manuscript. All authors contributed to the article and approved the submitted version.
