# Supplementary material for: Large-scale cross-sectional online survey on patient-neurologist communication, burden of disease assessment and disease monitoring in people with multiple sclerosis
Source: Front Neurol. 2023 Jan 4;13:1093352. doi: 10.3389/fneur.2022.1093352 (PMC9848394; doi:10.3389/fneur.2022.1093352)
Supplement: Supplementary file 1 [file Table_1.DOCX]

**Supplement**

Supplementary Tables

Supplementary Table S1: Questionnaire (as published previously [1])

**Demographic Questions**

| **Q01** | **How old are you?** |
| --- | --- |
|  | Numerical Input |
| **Q02** | **You are:** |
|  | Male |
|  | Female |
|  | Diverse |
| **Q03** | **Do you currently live in Germany?** |
|  | Yes |
|  | No |

**Patient journey**

| **Q04** | **Do you have multiple sclerosis (MS)?** |
| --- | --- |
|  | Yes |
|  | No |
| **Q05** | **How long ago did your first MS symptoms start?** |
|  | Less than 1 year |
|  | 1-2 years |
|  | 2-5 years |
|  | 5-10 years |
|  | 10-15 years |
|  | 15-20 years |
|  | More than 20 years. |
| **Q06** | **How long ago was your MS diagnosed?** |
|  | Less than 1 year |
|  | 1-2 years |
|  | 2-5 years |
|  | 5-10 years |
|  | 10-15 years |
|  | 15-20 years |
|  | More than 20 years |
| **Q07** | **Do you know the course of your MS?**  **Information: MS is often divided into three disease courses.**   - Relapsing-remitting MS (RRMS) progresses in relapses, which means that symptoms may last for days or weeks, and symptoms often resolve after the relapse ends. - Secondary progressive MS (SPMS) often develops from relapsing-remitting MS after years of disease. The symptoms do no longer occur (only) caused by relapses, but increase gradually, usually over at least 6 months. - Primary progressive MS (PPMS) in which symptoms increase continuously. As a rule, no relapses occur. |
|  | Yes, relapsing-remitting MS (RRMS). |
|  | Yes, secondary progressive MS (SPMS, gradual MS after relapsing onset, relapses may still occur). |
|  | Yes, primary progressive MS (PPMS). |
|  | No, I do not know my disease course. |
| **Q08** | **How long have you had a diagnosis of secondary progressive MS (SPMS)?** |
|  | Less than 1 year. |
|  | 1-2 years |
|  | 2-5 years |
|  | 5-10 years |
|  | 10-15 years |
|  | 15-20 years |
|  | More than 20 years |
| **Q09** | **Was the EDSS score assessed at your last visit?**  **Information: The Expanded Disability Status Scale (EDSS) is a score that describes the limitation/disability due to MS.** |
|  | Yes |
|  | No |
| **Q0902** | **Do you know what the EDSS score was at your last visit?** |
|  | Yes, I know my score. |
|  | My doctor told me, but I cannot remember. |
|  | My doctor did not tell me. |
| **Q0903** | **Please indicate what the EDSS score was at your last visit.** |
|  | Numerical input |
| **If Q9 was “No”: Q10** | **How would you yourself describe the severity of your MS?** |
|  | No disability |
|  | Minimal disability |
|  | Moderate disability (unrestricted walking distance) |
|  | Able to walk 500 m without assistance |
|  | Able to walk 200 m without assistance |
|  | Unilateral assistance required for 100 m |
|  | Walking distance restricted up to 5 m, predominantly restricted to wheelchair. |
|  | Predominantly restricted to wheelchair, chair, or bed. |

**Drug treatment**

| **Q11** | **Are you currently taking medication for immunotherapy for MS?** | | | | | | |
| --- | --- | --- | --- | --- | --- | --- | --- |
|  | Yes | | | | | | |
|  | No | | | | | | |
| **Q1102** | **What medications are you currently taking for immunotherapy for your MS?** | | | | | | |
|  | Oral medications. | | | | | | |
|  | Injectables. | | | | | | |
|  | Infusions (administration in hospital, by specialist). | | | | | | |
| **Q1103** | **How long have you been taking the current medication?** | | | | | | |
|  | Less than 6 months. | | | | | | |
|  | Between 6 and 12 months | | | | | | |
|  | 1-2 years | | | | | | |
|  | 2-5 years | | | | | | |
|  | More than 5 years | | | | | | |
| **Q12** | **Are you currently taking medication to treat symptoms of MS?** | | | | | | |
|  | Yes | | | | | | |
|  | No | | | | | | |
| **Q13** | **How long have you been taking medications for the following MS symptoms?** | | | | | | |
|  | | Does not apply | Less than 6 months | Between 6 and 12 months | 1-2 years | 2-5 years | More than 5 years |
| Spasticity | |  |  |  |  |  |  |
| Bladder disorder | |  |  |  |  |  |  |
| Gait disturbances / impaired walking ability | |  |  |  |  |  |  |
| Pain/discomfort | |  |  |  |  |  |  |
| Depression | |  |  |  |  |  |  |
| Fatigue (abnormal fatigability / exhaustion) | |  |  |  |  |  |  |
| **Q14** | **Are you currently using non-drug therapies?** | | | | | | |
|  | Yes | | | | | | |
|  | No | | | | | | |
| **Q15** | **What non-pharmacologic symptomatic therapies are you currently using? (Multiple choice)** | | | | | | |
|  | Physiotherapy | | | | | | |
|  | Ergotherapy | | | | | | |
|  | Logopedics | | | | | | |
|  | Neuropsychological training (cognitive training, training of concentration and memory etc.) | | | | | | |
|  | Psychotherapy | | | | | | |

**Relapse Management**

| **Q16** | **Have you had a relapse / relapses (new symptoms or worsened symptoms independent of infections / vaccinations) in the last 6 months?** |
| --- | --- |
|  | Yes |
|  | No |
| **Q17** | **How many relapses occurred in the last 6 months?** |
|  | Numeric input |
| **Q18** | **Please indicate how well you have recovered from your last relapse?** |
|  | Complete (100%) |
|  | Almost complete (75%) |
|  | Partial (50%) |
|  | A little bit (25%) |
|  | Not at all (0%) |
| **Q19** | **Have you experienced any of the following symptoms in the past 6 months? (Multiple Choice)** |
|  | Vision impairment |
|  | Muscle weakness and stiffness |
|  | Problems with walking and moving |
|  | Problems with coordination and balance |
|  | Pain |
|  | Numbness or tingling |
|  | Problems with bladder or bowel control |
|  | Speech problems |
|  | Problems with concentrating and remembering |
|  | Fatigue (abnormal fatigability / exhaustion) |
| **Q19a (for each symptom chosen in Q19)** | **Has this symptom occurred with every episode you have had in the last 6 months?** |
|  | Yes |
|  | No |
| **Q19b (for each symptom chosen in Q19)** | **Did the symptom go away after it appeared or was it present most of the time?** |
|  | The symptom is present most of the time. |
|  | The symptom disappeared again after its appearance. |
| **Q19c (for each symptom chosen in Q19)** | **Did the symptom improve, stay the same, or get worse?** |
|  | Improved |
|  | Stayed the same |
|  | Got worse |

**Disease burden: Outside relapse periods**

| **Q20** | **In the past 6 months, outside of a relapse, how much have your MS symptoms affected you in your daily life in the following situations?** | | | | | |
| --- | --- | --- | --- | --- | --- | --- |
|  | | Not at all | A little | Moderate | Severe | Not possible on my own due to my MS |
| Mobility (moving) | |  |  |  |  |  |
| Washing, bathing, and dressing | |  |  |  |  |  |
| Performing daily tasks (e.g., housework or driving) | |  |  |  |  |  |
| Work (paid or voluntary) | |  |  |  |  |  |
| Practising hobbies or leisure activities | |  |  |  |  |  |
| Being intimate or having sex | |  |  |  |  |  |
| Emotional experiences (e.g., feeling happy or anxious or worried). | |  |  |  |  |  |
| **Q21** | **Which of the symptoms experienced at any time during MS do you rate as most bothersome for you and your daily activities? Please select only one of the following symptoms.** | | | | | |
|  | Vision impairment | | | | | |
|  | Muscle weakness | | | | | |
|  | Problems with walking and moving | | | | | |
|  | Problems with coordination and balance | | | | | |
|  | Pain | | | | | |
|  | Numbness or tingling | | | | | |
|  | Problems with bladder or bowel control | | | | | |
|  | Speech problems | | | | | |
|  | Problems with concentrating and remembering | | | | | |
|  | Fatigue (abnormal fatigability / exhaustion) | | | | | |
|  | Problems with intimacy/sex | | | | | |
| **Q22** | **Is (or was) your job impaired because of your MS?** | | | | | |
|  | No, I am not working for reasons that are not related to my MS. | | | | | |
|  | No, my MS does not interfere with my job. | | | | | |
|  | Yes, I had to reduce my working hours. | | | | | |
|  | Yes, I had to reduce my working hours and then take early retirement. | | | | | |
|  | Yes, I had to quit my previous job and take early retirement because of my MS. | | | | | |
|  | Yes, I am considering cutting back professionally. | | | | | |
|  | Yes, I had to retrain. | | | | | |
| **Q23** | **For your current immunotherapy, have specific treatment goals been discussed with your physician (such as stabilization or improvement of disease with respect to relapses, MRI findings, disability progression, walking distance)?** | | | | | |
|  | Yes | | | | | |
|  | No | | | | | |
| **Q24** | **Which of the following symptom(s) has/have continuously worsened in the last 12 months, independent of relapses? (Multiple choice)** | | | | | |
|  | None | | | | | |
|  | Vision impairment | | | | | |
|  | Muscle weakness and stiffness | | | | | |
|  | Problems with walking | | | | | |
|  | Problems with coordination and balance | | | | | |
|  | Pain | | | | | |
|  | Numbness or tingling | | | | | |
|  | Problems with bladder or bowel control | | | | | |
|  | Speech problems | | | | | |
|  | Problems with concentrating and remembering | | | | | |
|  | Fatigue (abnormal fatigability / exhaustion) | | | | | |
| **Q24a (for each symptom chosen in Q24)** | **Have you already talked to your doctor about this?** | | | | | |
|  | Yes, my doctor has asked me about it | | | | | |
|  | Yes, I have asked my doctor about it | | | | | |
|  | No, I do not want to talk to my doctor about it | | | | | |
|  | No, there has not yet been an opportunity | | | | | |
| **Q24b (for each symptom chosen in Q24)** | **What consequence was drawn from this? (Multiple choice)** | | | | | |
|  | Closer controls | | | | | |
|  | Further examinations (e.g., magnetic resonance tomography, walking distance, nerve measurements) | | | | | |
|  | Change of therapy | | | | | |
|  | None (Exclusive) | | | | | |
| **Q25** | **Which of these symptoms was most disabling for you in your daily life? Please select only one of the following limitations/symptoms.** | | | | | |
|  | Vision impairment | | | | | |
|  | Muscle weakness and stiffness | | | | | |
|  | Problems with walking | | | | | |
|  | Problems with coordination and balance | | | | | |
|  | Pain | | | | | |
|  | Numbness or tingling | | | | | |
|  | Problems with bladder or bowel control | | | | | |
|  | Speech problems | | | | | |
|  | Problems with concentrating and remembering | | | | | |
|  | Fatigue (abnormal fatigability / exhaustion) | | | | | |

**Diagnostics/controls at the doctor**

| **Q26** | **What aspects does your neurologist regularly record during your follow-up visits? Please select all that apply.** |
| --- | --- |
|  | Walking distance (reported/recorded on site or from everyday life). |
|  | Test for hand function (e.g., Nine Hole Peg Test) |
|  | Magnetic resonance imaging (MRI) findings |
|  | (Questionnaire for) cognition/cognition test |
|  | (Questionnaire on) Fatigue (abnormal fatigability / exhaustion) |
|  | (Questionnaire on) Depression |
|  | Quality of life questionnaire |
|  | None of the above |
| **Q27** | **How often do you have a magnetic resonance imaging (MRI) scan?** |
|  | Semiannual |
|  | Annual |
|  | Every 2 years |
|  | More than every 2 years |
| **Q28** | **Does the magnetic resonance imaging (MRI) scan involve the administration of a contrast agent?** |
|  | Yes, always. |
|  | Yes, frequently. |
|  | No, never. |
| **Q29** | **Does your doctor discuss the magnetic resonance imaging (MRI) scan (results) images with you?** |
|  | Yes |
|  | No |
| **Q30** | **Do you always see the same radiologist?** |
|  | Yes, always. |
|  | No |
| **Q31** | **How important is magnetic resonance imaging (MRI) monitoring to you personally?** |
|  | Important (e.g., it gives me security whether everything is stable). |
|  | Unimportant (e.g., it is just a picture that has no meaning to me without symptoms). |
|  | I do not know / No opinion. |
| **Q32** | **Would you like to learn more about the role of magnetic resonance imaging (MRI) or MRI scans in your condition?** |
|  | Yes |
|  | No |
|  | I do not know / No opinion. |

**Patient Symptom Management**

| **Q33** | **Which of the following do you use to monitor your health? (Multiple choice)** |
| --- | --- |
|  | Smartwatch (such as Apple Watch or Fitbit) |
|  | Pedometer |
|  | Diary (electronic, paper) |
|  | App(s) namely: _______________ |
|  | None (Exclusive) |
| **Q34** | **How often do you use these objects to monitor/record your health?** |
|  | Regularly |
|  | Irregularly |
| **Q35** | **Which functions of apps do you use? (Multiple choice)** |
|  | Document function |
|  | Information search |
|  | Communication |
|  | Detection of certain body functions |
|  | Memory functions |
|  | Other |
| **Q36** | **Do these functions play a role in your conversation with your neurologist?** |
|  | Yes |
|  | No |

**References**

1. Bayas, A.; Schuh, K.; Christ, M. Self-assessment of people with relapsing-remitting and progressive multiple sclerosis towards burden of disease, progression, and treatment utilization – results of a large-scale cross-sectional online survey (MS Perspectives). *Mult Scler Relat Disord* **2022**, *68*, 104166, doi:10.1016/j.msard.2022.104166.

Supplementary Figures

A


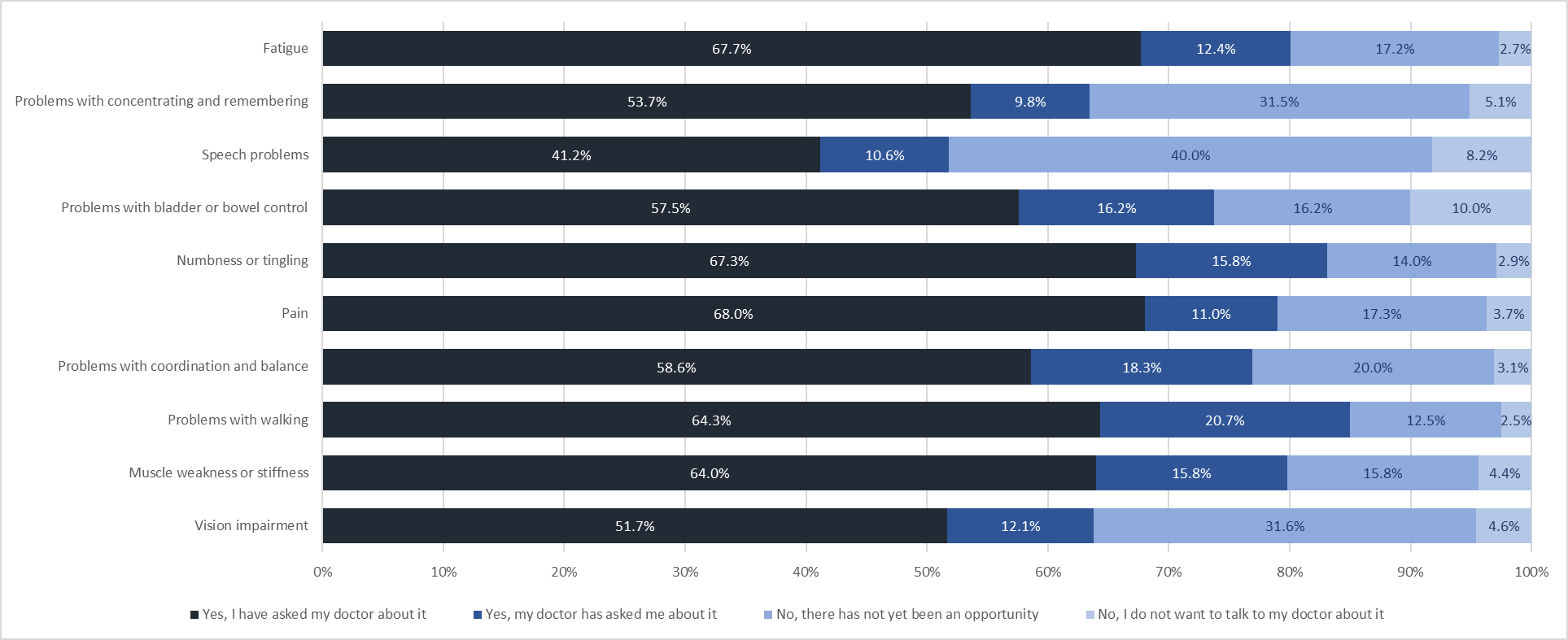


B


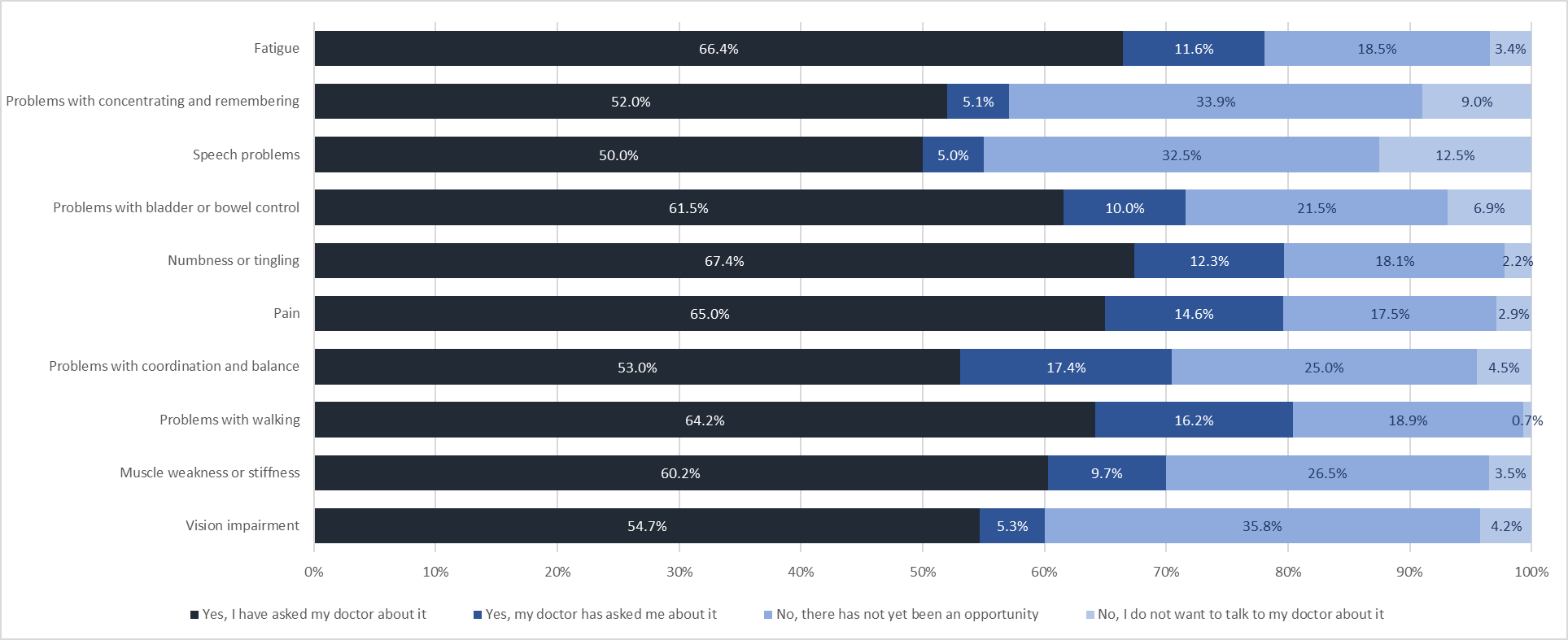


C


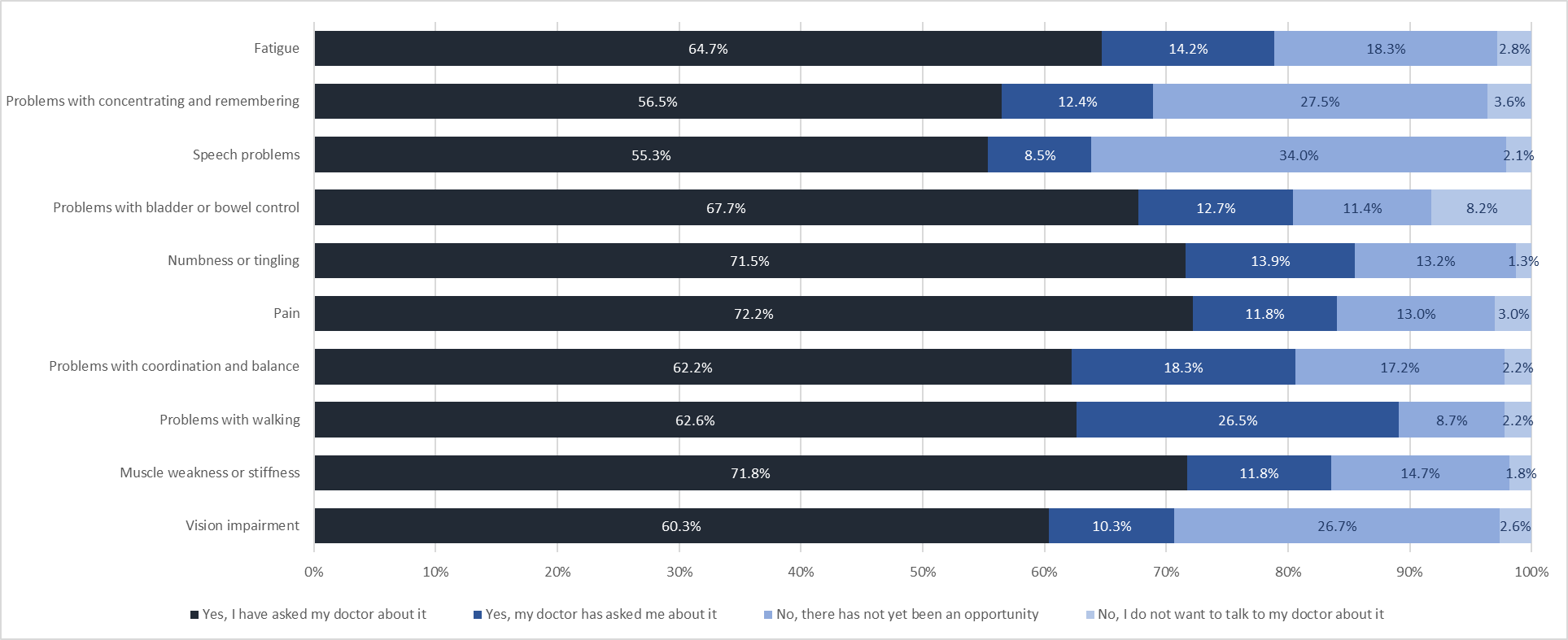


Supplementary Figure S1: Reporting of symptoms worsened in the last 12 months independent of relapses by treatment type: A) oral medications; B) injectables; C) infusions.

A


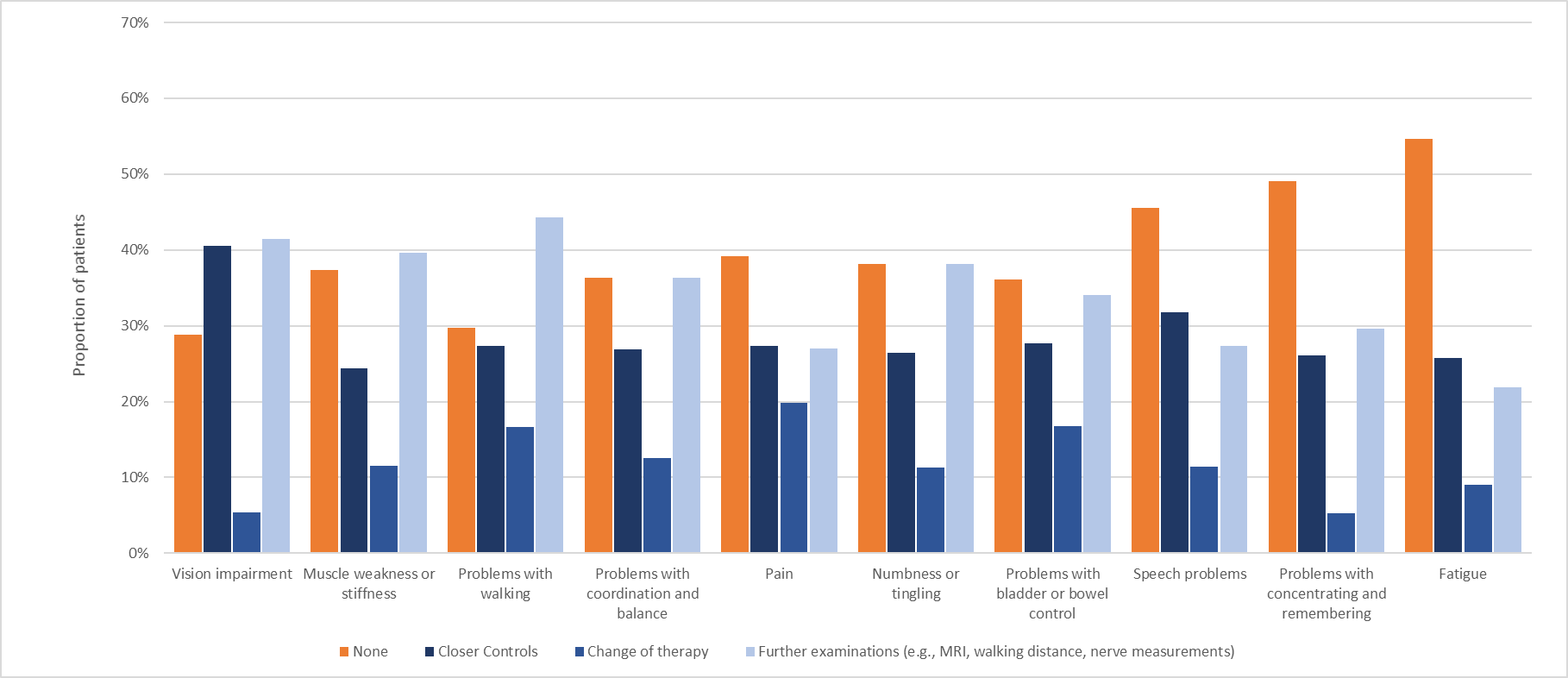


B


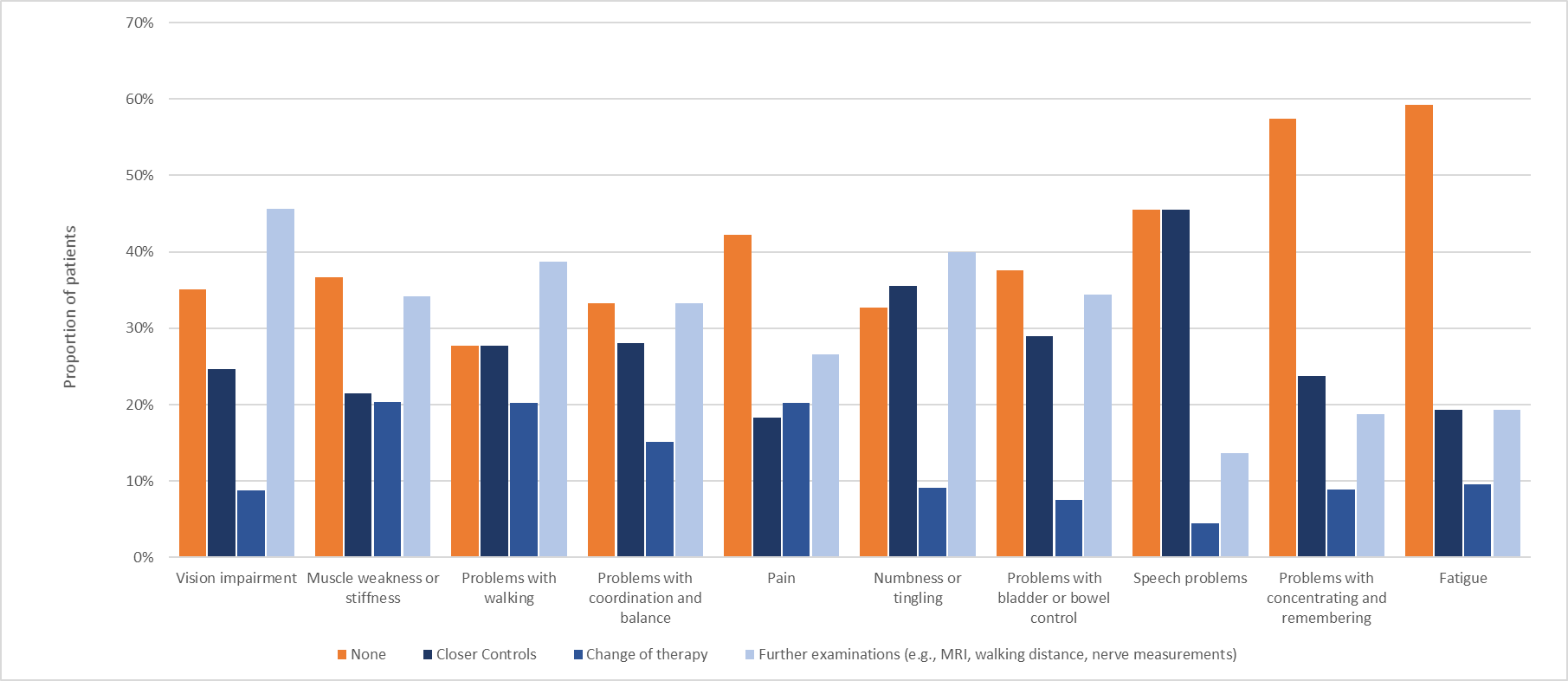


C


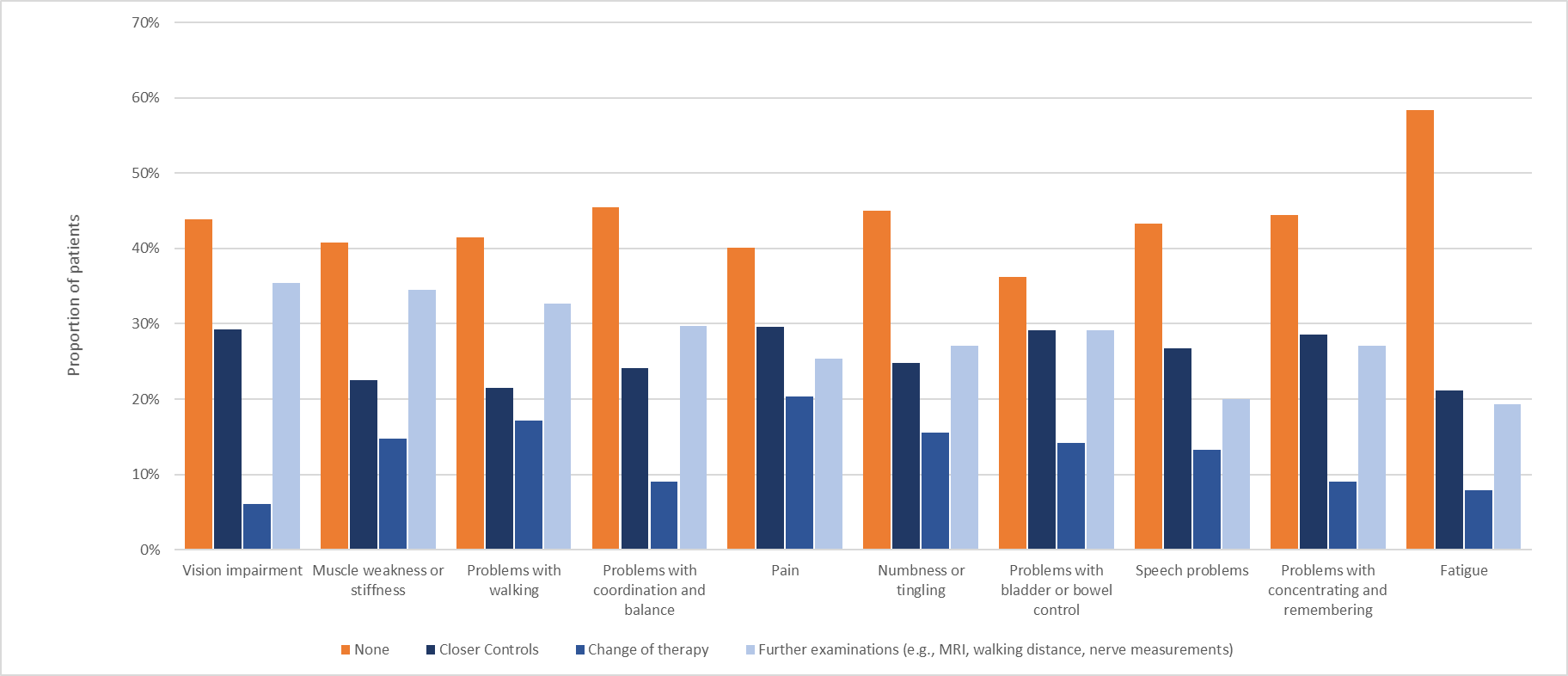


Supplementary Figure S2: Actions taken regarding symptoms worsened in the last 12 months independent of relapses by treatment type: A) oral medications; B) injectables; C) infusions
